# Supplementary material for: Computational Methods for Understanding the Selectivity and Signal Transduction Mechanism of Aminomethyl Tetrahydronaphthalene to Opioid Receptors
Source: Molecules. 2022 Mar 28;27(7):2173. doi: 10.3390/molecules27072173 (PMC9000250; doi:10.3390/molecules27072173)
Supplement: Supplementary file 1 [file molecules-27-02173-s001.zip › molecules-1642440 supplementary 3.28 .pdf]

molecules

MDPI

Computational Methods for Understanding the Selectivity and Signal Transduction Mechanism of Aminomethyl Tetrahydronaphthalene to Opioid Receptors

Peng Xie [1,2], Junjie Zhang [1], Baiyu Chen [1], Xinwei Li [1], Wenbo Zhang [2], Mengdan Zhu [1], Wei Li [1], Jianqi Li [2,*] and Wei Fu [1,*]

1 School of Pharmacy, Fudan University, Shanghai 201301, P. R. China;
2 Novel Technology Center of Chemical Pharmaceutics, China State Institute of Pharmaceutical Industry, Shanghai 201203, P. R. China.
* Correspondence: wfu@fudan.edu.cn

Supporting Material

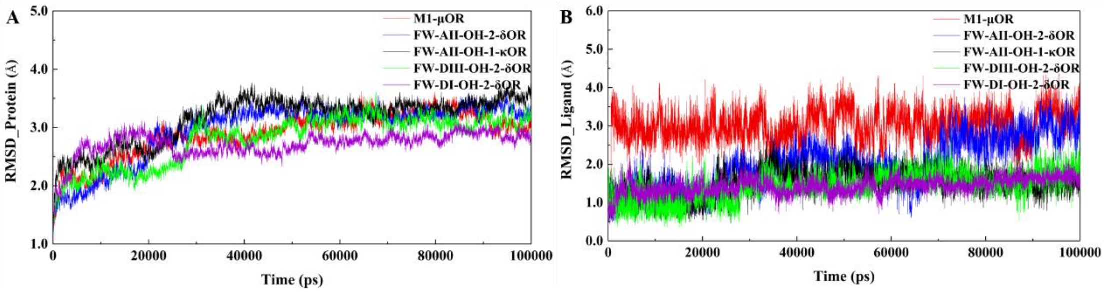

Figure S1. The RMSD values of the protein (**A**) and ligand (**B**) in the simulation time.

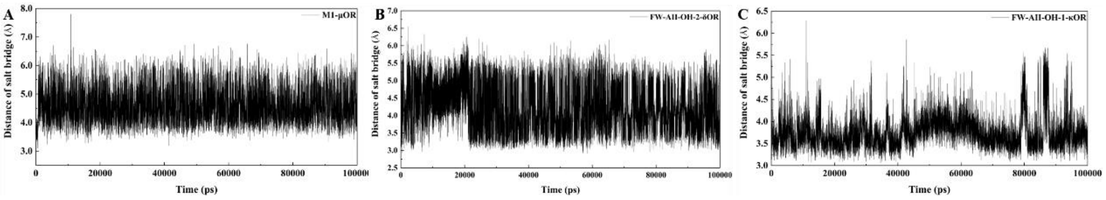

Figure S2. The distances of salt bridges between $D^{3.32}$ and amine groups in **M1** (**A**), **FW-AII-OH-2** (**B**) and **FW-AII-OH-1** (**C**).

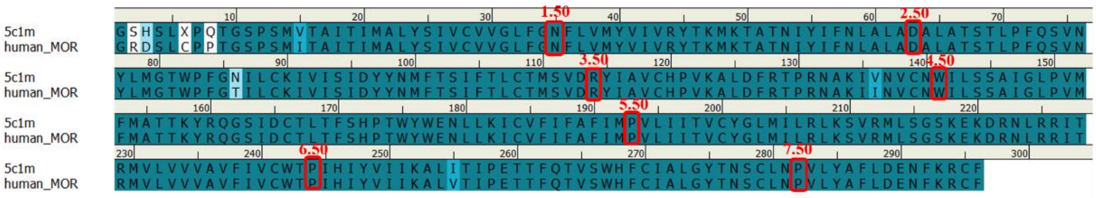

Figure S3. Result of sequence alignment between human MOR and mouse MOR.

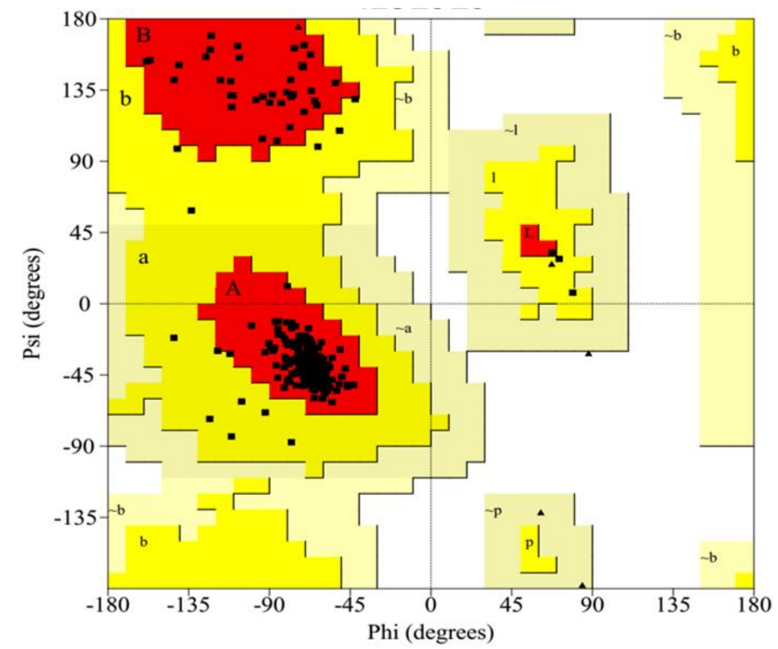

Plot statistics

Residues in most favoured regions [A,B,L]	257	94.8%
Residues in additional allowed regions [a,b,l,p]	14	5.2%
Residues in generously allowed regions [~a,~b,~l,~p]	0	0.0%
Residues in disallowed regions	0	0.0%
	----	-------
Number of non-glycine and non-proline residues	271	100.0%
Number of end-residues (excl. Gly and Pro)	1	
Number of glycine residues (shown as triangles)	11	
Number of proline residues	13	

Total number of residues	296	

Figure S4. Main Ramachandran plot of active MOR homology structure.

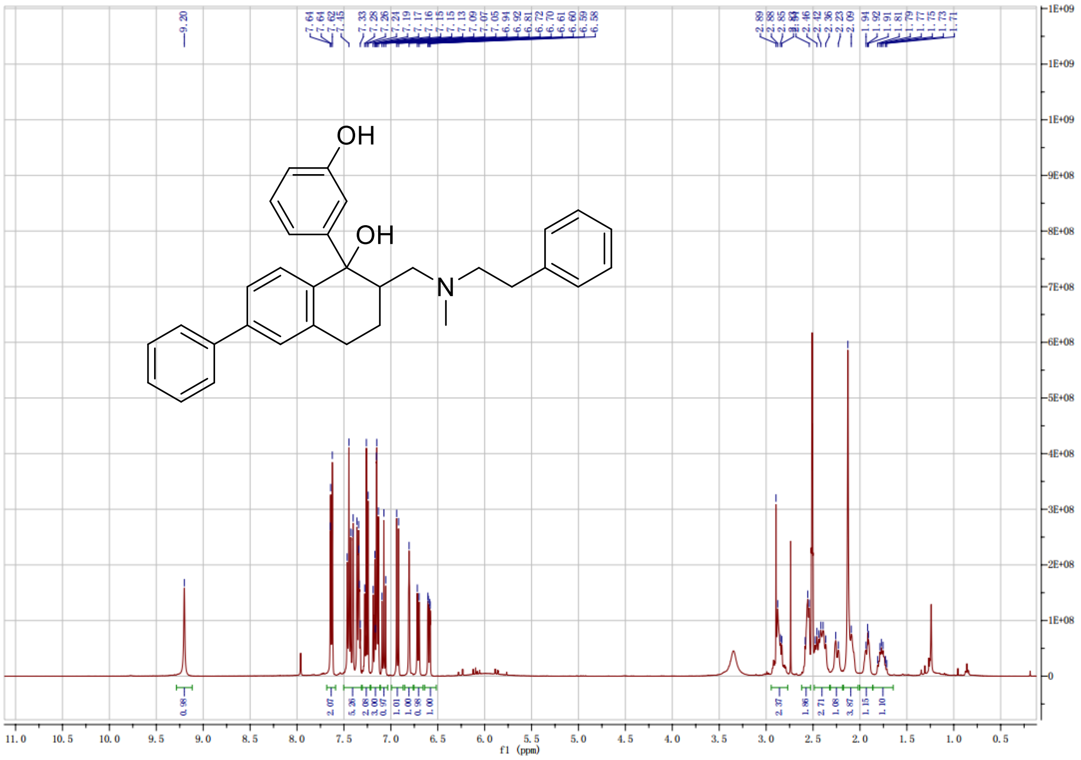

Figure S5. ^{1}HNMR Spectroscopic Data of FW-DIII-OH-2.

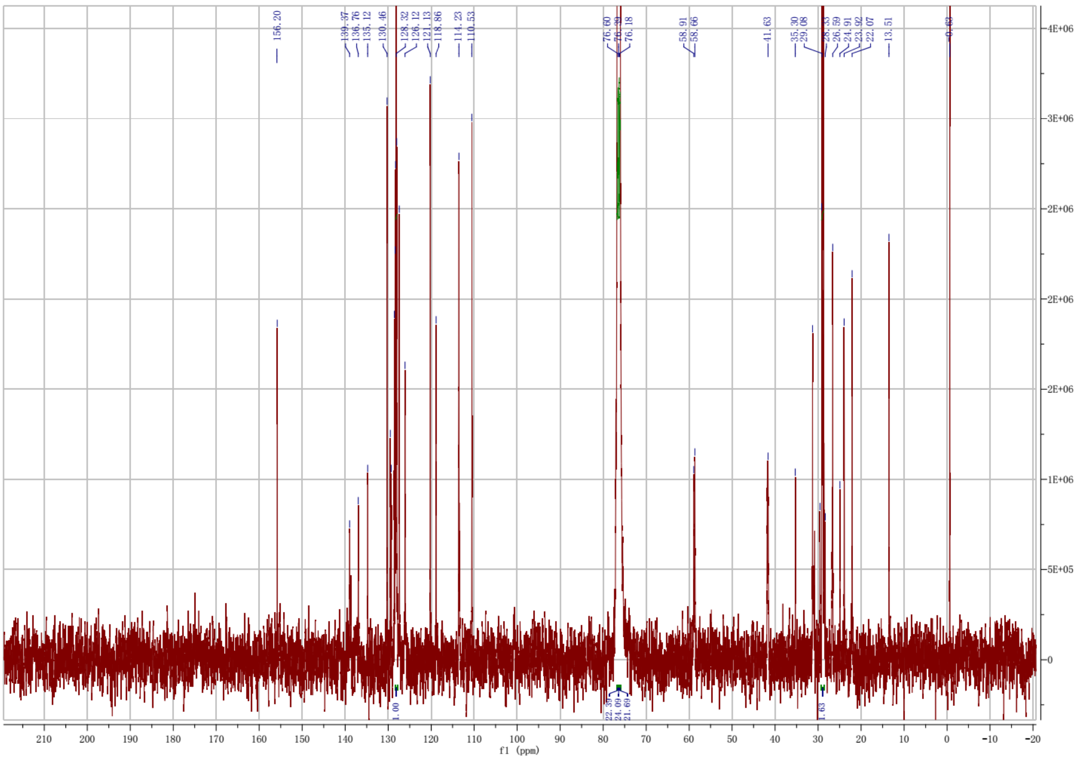

Figure S6. ^{13}CNMR Spectroscopic Data of FW-DIII-OH-2.

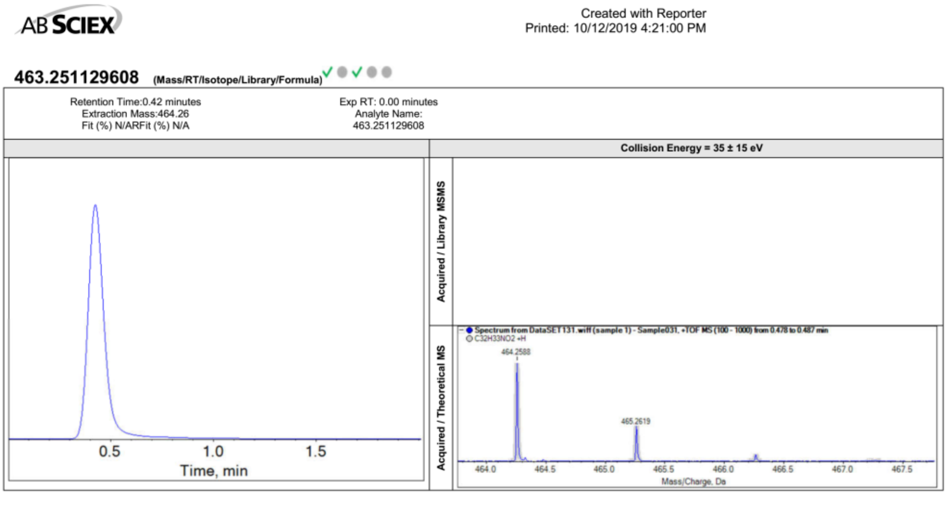

	Compound Name (Library Hit)	Score	Formula	Intensity	Threshold	Expected m/z	Found at m/z	Error (ppm)	Expected RT (min)	Found RT (min)	RT Delta (min)	Isotope Diff (%)	Library Score(%)
	463.251129608(FW-DIII-OH-2)	98%	C32H33NO2	12709844	5	464.2584	464.2588	0.8	0.00	0.42	0.42	0.8%	N/A

Figure S7. HRMS Spectroscopic Data of FW-DIII-OH-2.

FW-DI-OH-2

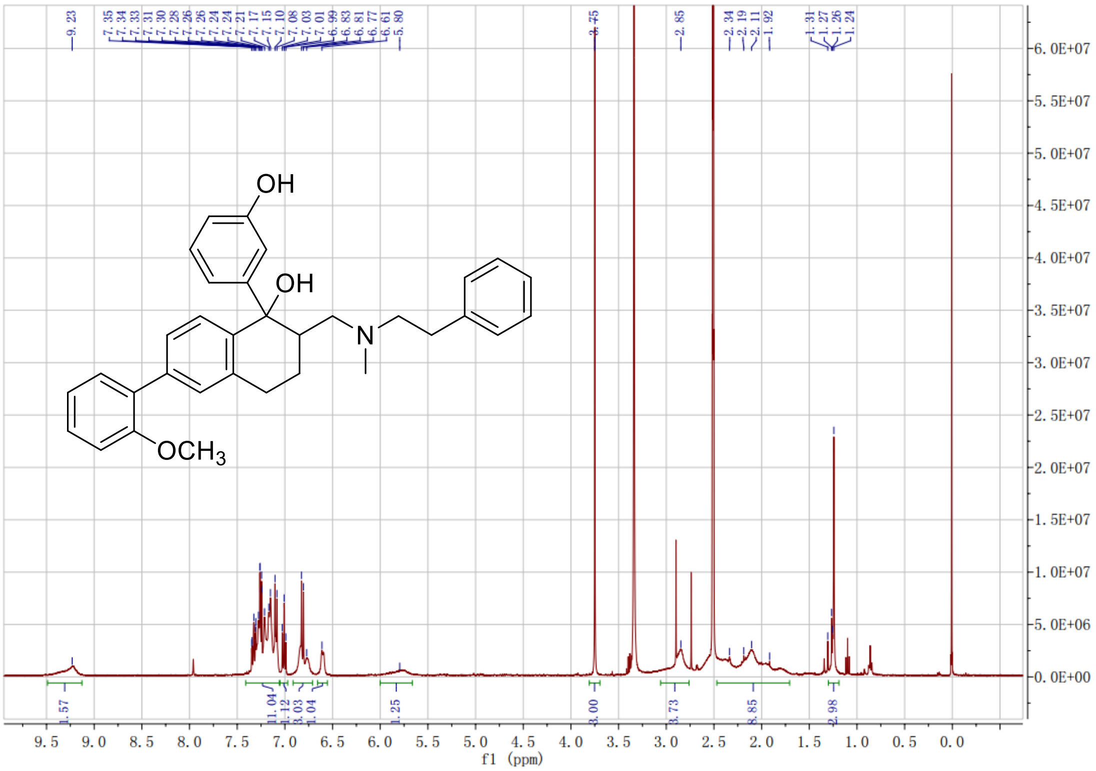

Figure S8. ^{1}HNMR Spectroscopic Data of FW-DI-OH-2.

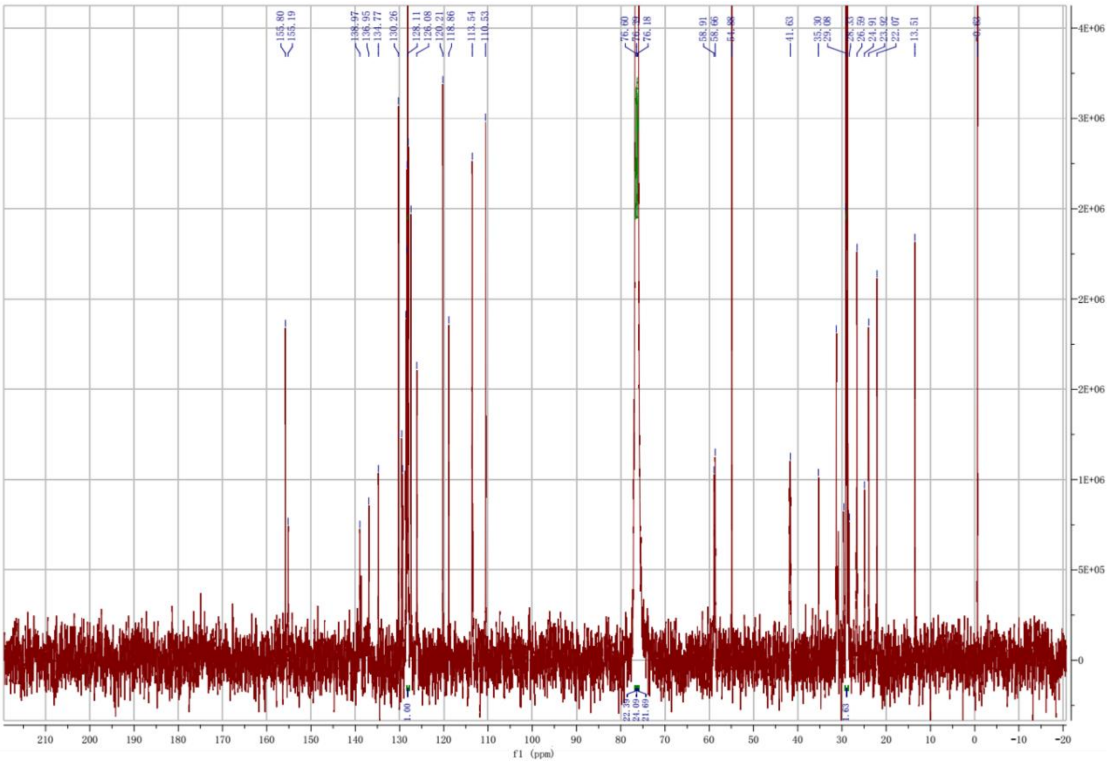

Figure S9. ^{13}CNMR Spectroscopic Data of FW-DI-OH-2.

AB SCIEX

Created with Reporter
Printed: 10/12/2019 4:22:06 PM

493.261694328 (Mass/RT/Isotope/Library/Formula)

Retention Time:0.42 minutes
Extraction Mass:494.27
Fit (%) N/ARFit (%) N/A

Exp RT: 0.00 minutes
Analyte Name:
493.261694328

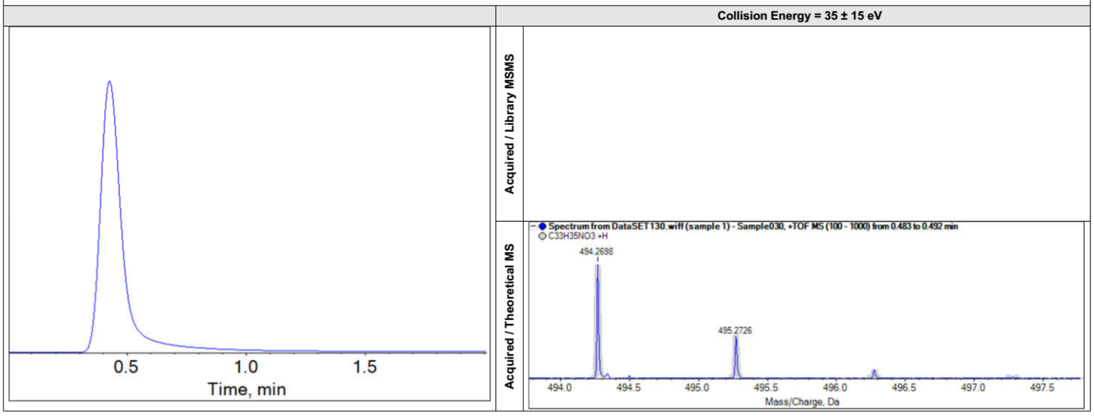

	Compound Name (Library Hit)	Score	Formula	Intensity	Threshold	Expected m/z	Found at m/z	Error (ppm)	Expected RT (min)	Found RT (min)	RT Delta (min)	Isotope Diff (%)	Library Score(%)
	493.261694328(FW-DI-OH-2)	96%	C33H35NO3	15301187	5	494.2690	494.2698	1.7	0.00	0.42	0.42	0.7%	N/A

Figure S10. HRMS Spectroscopic Data of FW-DI-OH-2.

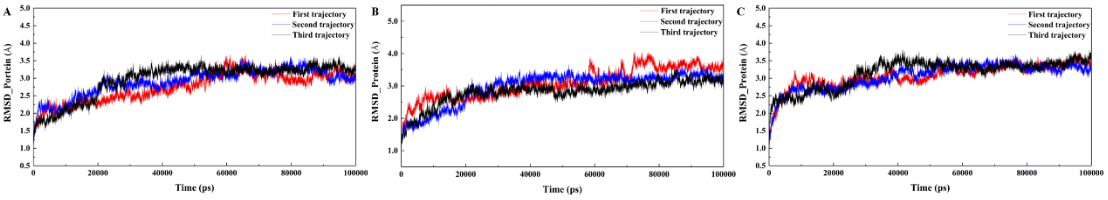

Figure S11. The RMSD values of the proteins (**A**). **M1-MOR** complex, (**B**). **FW-AII-OH-2**-DOR complex, (**C**). **FW-AII-OH-1**-KOR complex.

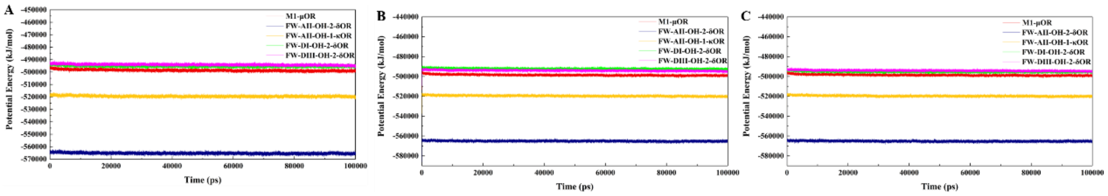

Figure S12. The time evolved potential energy profile of complexes (A). First trajectory, (B) Second trajectory, (C) Third trajectory.

M1-MOR complex

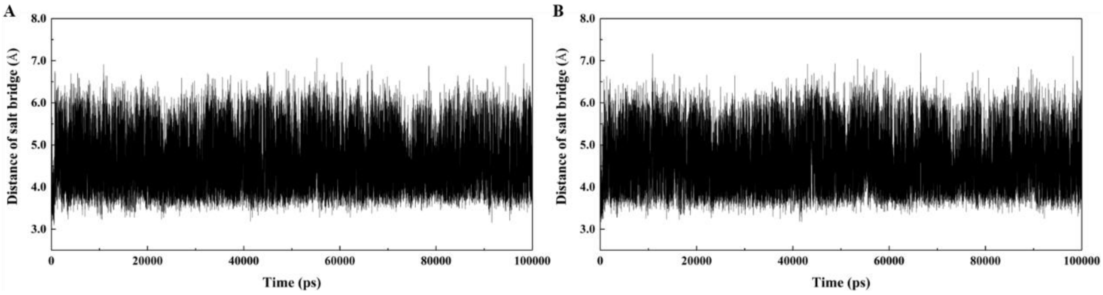

Figure S13. The distances of salt bridges between $D^{3.32}$ and amine groups. (A). The second trajectory, (B). The third trajectory.

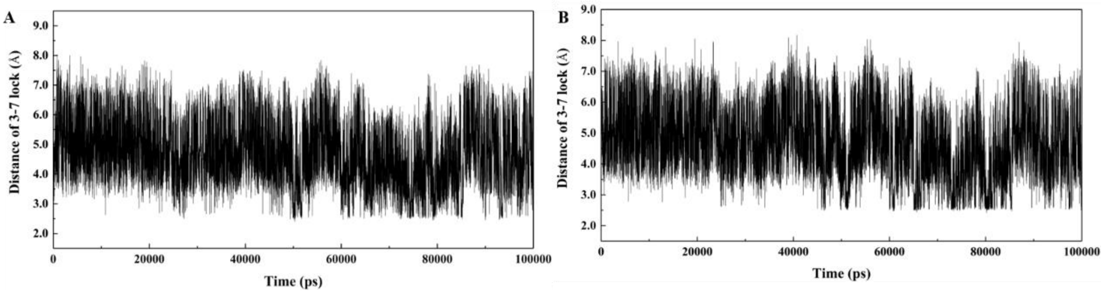

Figure S14. The distances of hydrogen bonds between $D^{3.32}$ and $Y^{7.43}$ (3-7lock). (A). The second trajectory, (B). The third trajectory.

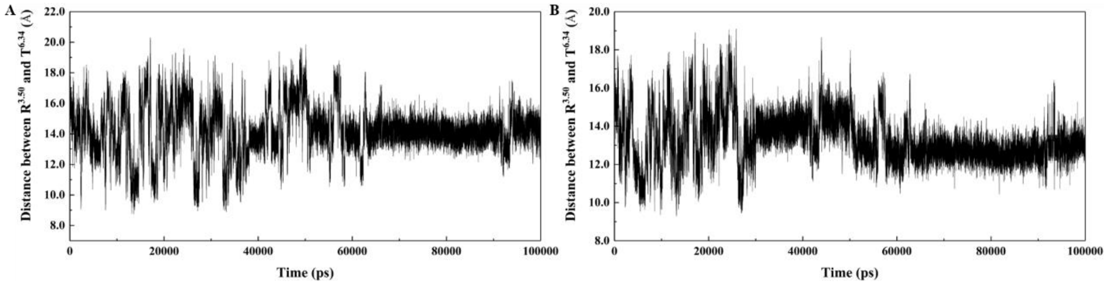

Figure S15. Variation of the distance between $R^{3.50}$ and $T^{6.34}$. (A). The second trajectory, (B). The third trajectory.

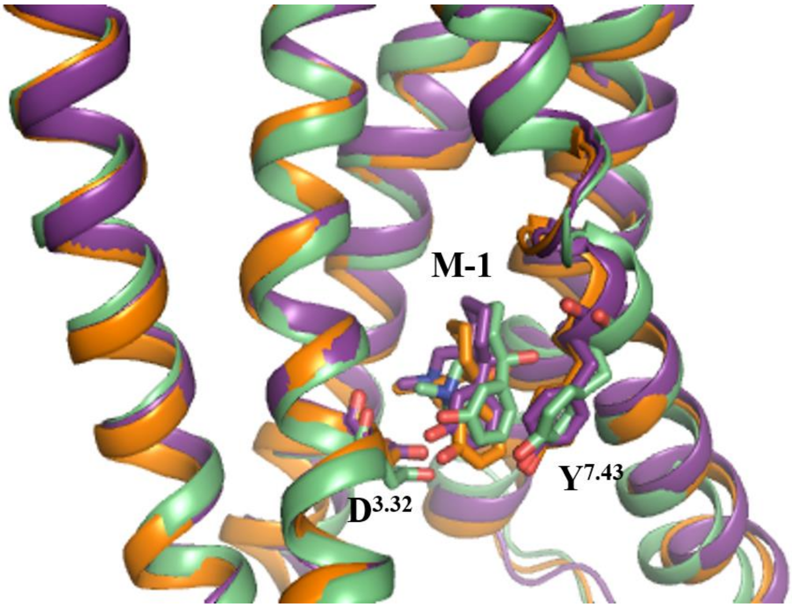

Figure S16. The alignment of binding mode in three trajectories.

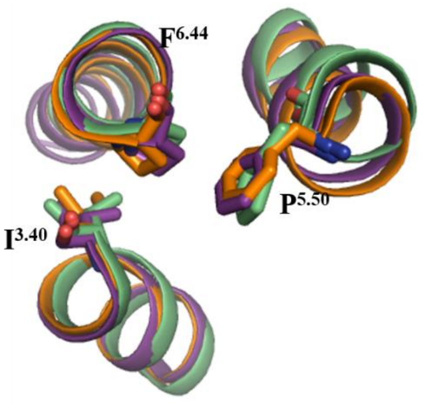

Figure S17. The alignment of $I^{3.40}$, $P^{5.50}$ and $F^{6.44}$ in three trajectories.

FW-AII-OH-2-DOR complex

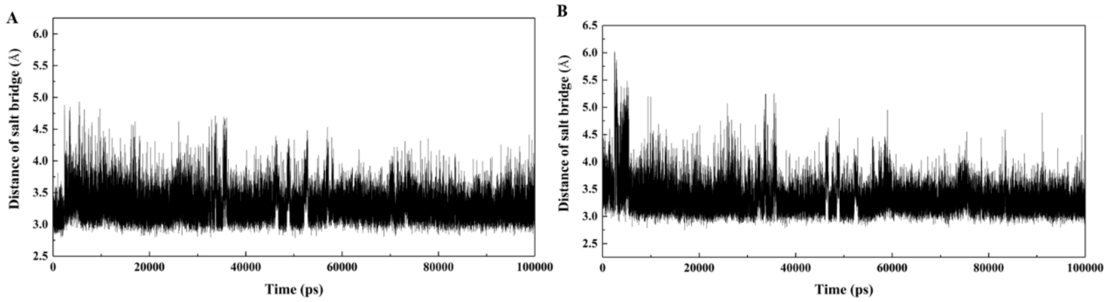

Figure S18. The distances of salt bridges between $D^{3.32}$ and amine groups. (A). The second trajectory, (B). The third trajectory.

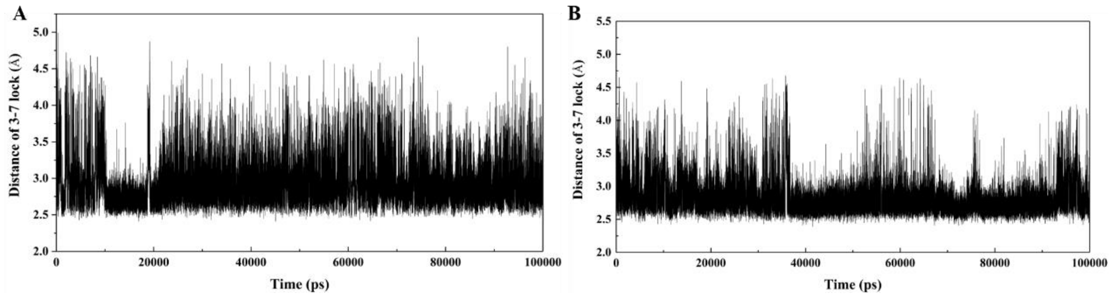

Figure S19. The distances of hydrogen bonds between $D^{3.32}$ and $Y^{7.43}$ (3-7lock). (A). The second trajectory, (B). The third trajectory.

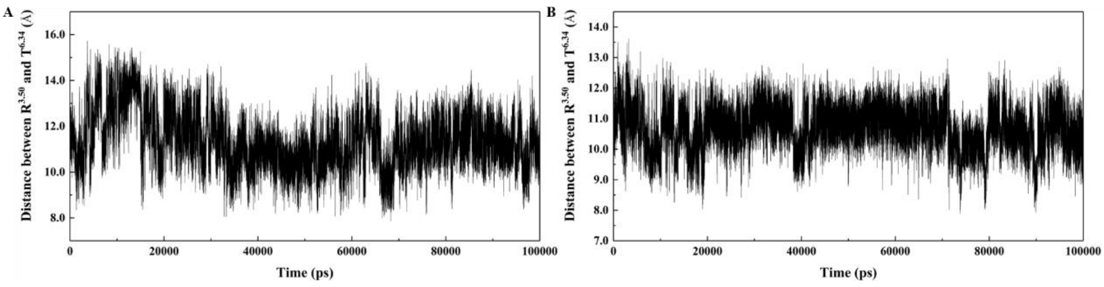

Figure S20. Variation of the distance between $R^{3.50}$ and $T^{6.34}$. (A). The second trajectory, (B). The third trajectory.

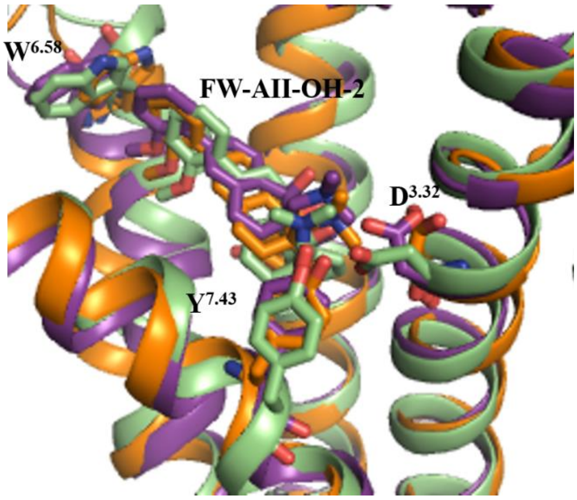

Figure S21. The alignment of binding mode in three trajectories.

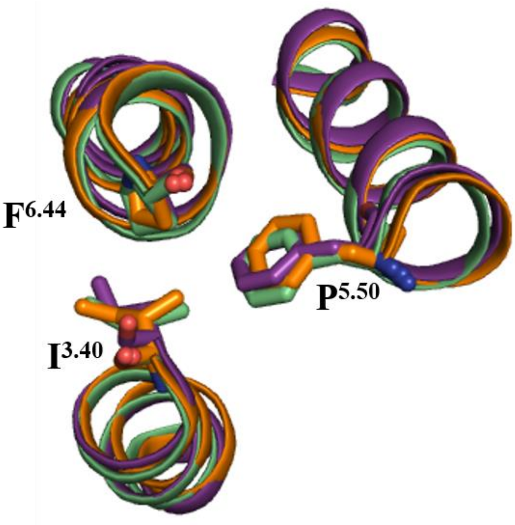

Figure S22. The alignment of $I^{3.40}$, $P^{5.50}$ and $F^{6.44}$ in three trajectories.

FW-AII-OH-1-KOR complex

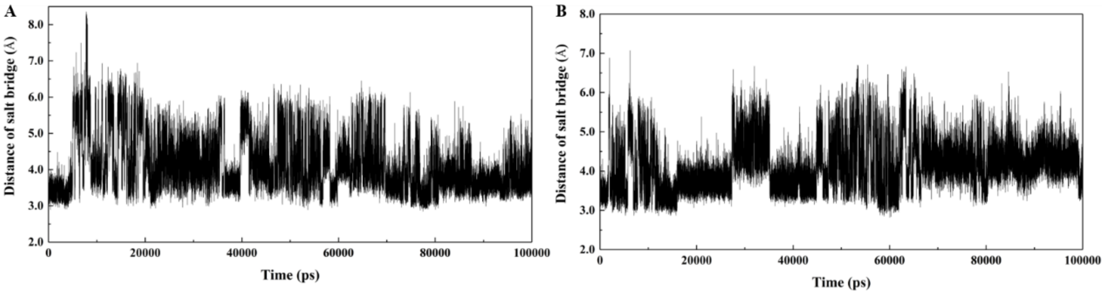

Figure S23. The distances of salt bridges between $D^{3.32}$ and amine groups. (A). The second trajectory, (B). The third trajectory.

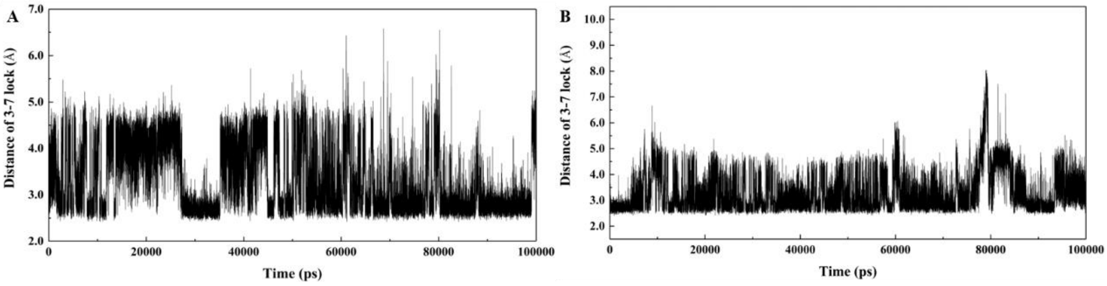

Figure S24. The distances of hydrogen bonds between $D^{3.32}$ and $Y^{7.43}$ (3-7lock). (A). The second trajectory, (B). The third trajectory.

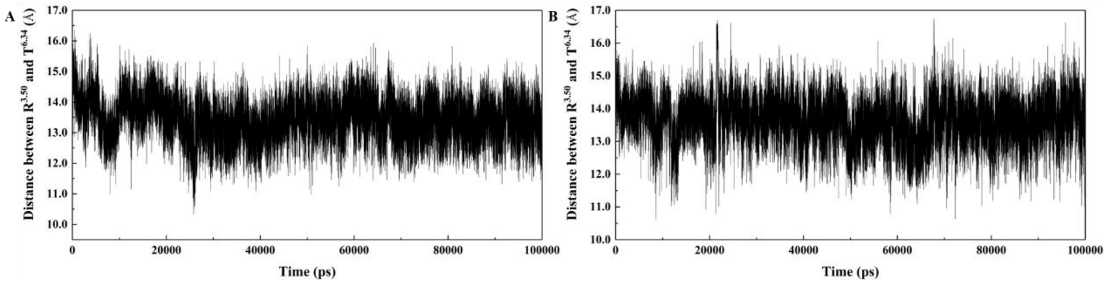

Figure S25. Variation of the distance between $R^{3.50}$ and $T^{6.34}$. (A). The second trajectory, (B). The third trajectory.

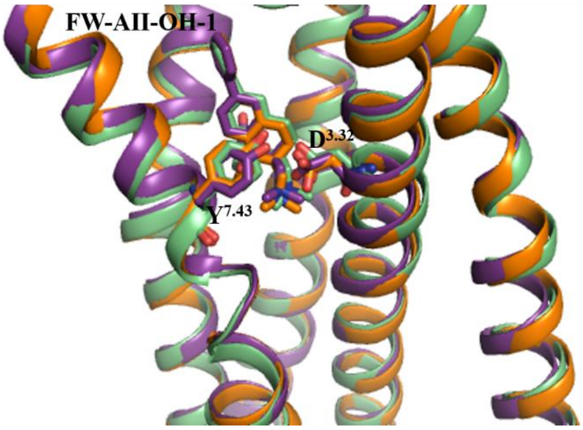

Figure S26. The alignment of binding mode in three trajectories.

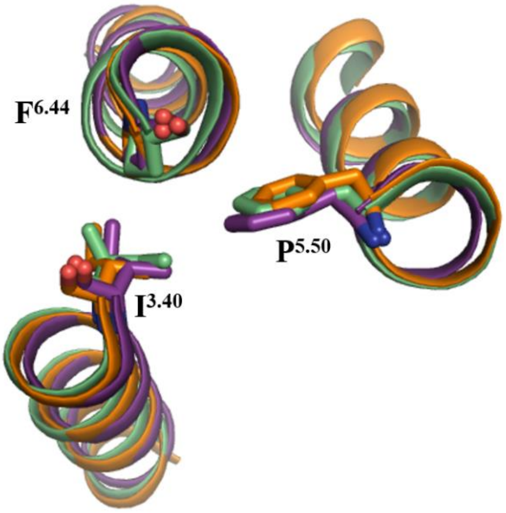

Figure S27. The alignment of $I^{3.40}$, $P^{5.50}$ and $F^{6.44}$ in three trajectories.

FW-DI-OH-2-DOR complex:

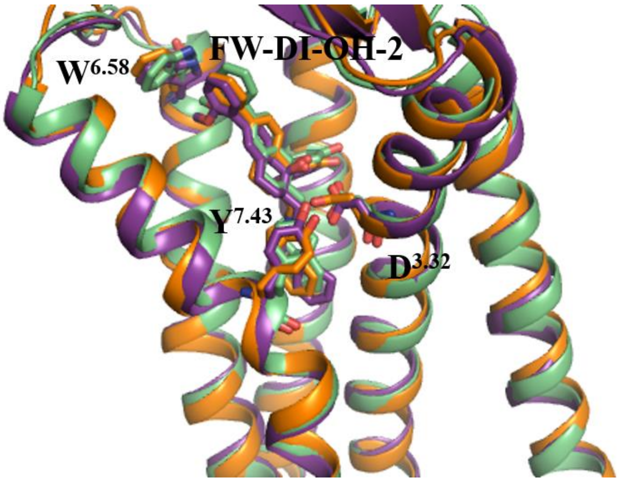

Figure S28. The alignment of binding mode in three trajectories.

FW-DIII-OH-2-DOR complex:

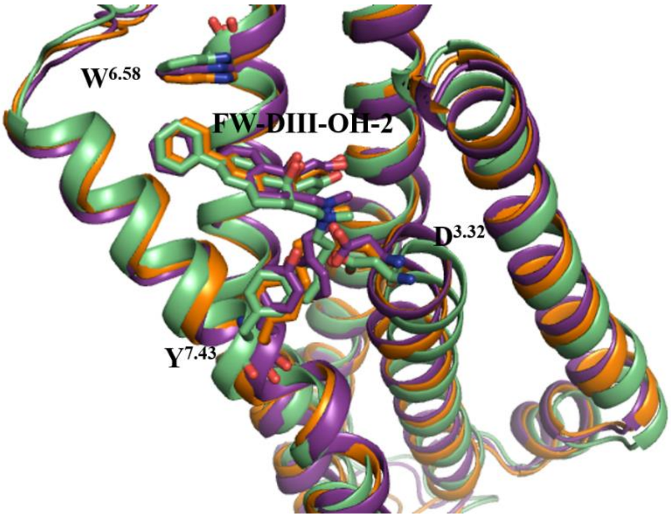

Figure S29. The alignment of binding mode in three trajectories.
